# Hypoxia biomarkers in squamous cell carcinoma of the uterine cervix

**DOI:** 10.1186/s12885-015-1828-2

**Published:** 2015-10-26

**Authors:** Christine Ellingsen, Lise Mari K. Andersen, Kanthi Galappathi, Einar K. Rofstad

**Affiliations:** Department of Radiation Biology, Group of Radiation Biology and Tumor Physiology, Institute for Cancer Research, Oslo University Hospital, Oslo, Norway

**Keywords:** Cervical carcinoma xenografts, Hypoxia, Pimonidazole, Hypoxia inducible factor-1α, Carbonic anhydrase IX

## Abstract

**Background:**

There is significant evidence that severe tumor hypoxia may cause resistance to chemoradiotherapy and promote metastatic spread in locally advanced carcinoma of the uterine cervix. Some clinical investigations have suggested that high expression of hypoxia-inducible factor-1α (HIF-1α) and/or its target gene carbonic anhydrase IX (CAIX) may be useful biomarkers of tumor hypoxia and poor outcome in cervical cancer. Here, we challenged this view by investigating possible associations between HIF-1α expression, CAIX expression, fraction of hypoxic tissue, and lymph node metastasis in experimental human tumors.

**Methods:**

Tumors of two cervical carcinoma xenograft lines (CK-160 and TS-415) were included in the study. Pimonidazole was used as a hypoxia marker, and tumor hypoxia, HIF-1α expression, and CAIX expression were detected by immunohistochemistry. Metastatic status was assessed by examining external lymph nodes in the inguinal, axillary, interscapular, and submandibular regions and internal lymph nodes in the abdomen and mediastinum.

**Results:**

Tissue regions staining positive for pimonidazole, HIF-1α, or CAIX were poorly colocalized, both in CK-160 and TS-415 tumors. The expression of HIF-1α or CAIX did not correlate with the fraction of hypoxic tissue in any of the two tumor lines. Furthermore, clinically relevant associations between HIF-1α or CAIX expression and lymph node metastasis were not found.

**Conclusion:**

Because significant associations between HIF-1α expression, CAIX expression, fraction of hypoxic tissue, and incidence of lymph node metastases could not be detected in any of two preclinical models of human cervical cancer, it is not realistic to believe that high expression of HIF-1α or CAIX can be useful biomarkers of tumor hypoxia and poor outcome in a highly heterogeneous disease like cervical carcinoma.

## Background

Carcinoma of the uterine cervix is the fourth leading cause of cancer-related death in women worldwide [1]. The prognosis is good for the earlier stages of the disease, but gets gradually worse with disease progression [2]. In the western world, concurrent chemoradiotherapy is the treatment of choice for locally advanced disease [3]. Significant cure rates are achieved with concurrent chemoradiotherapy, but at the expense of treatment-related toxicities [3, 4].

Primary tumor volume, histology, lymph node status, and stage of disease have been identified as important prognostic factors in cervical carcinoma [3]. The outcome of treatment can differ among individual patients in the same prognostic group, possibly because of differences in the physiological microenvironment of the tumor tissue [5]. Severe abnormalities in the tumor microenvironment, including low oxygen tension, elevated interstitial fluid pressure, and acidification of the extracellular space, have been shown to be associated with locoregional treatment failure and poor disease-free and overall survival rates [5–11]. Tumor oxygen tension is the most studied microenvironmental parameter, and there is strong evidence that severe hypoxia may cause resistance to chemoradiotherapy and promote metastatic dissemination in cervical carcinoma [12–15].

To improve the outcome of cervical cancer, reliable biomarkers that can identify patients with aggressive and treatment resistant tumors are needed [16]. Several endogenous proteins have been suggested as potential biomarkers of tumor hypoxia, including hypoxia-inducible factor-1α (HIF-1α, the α subunit of the transcription factor HIF-1) and downstream target genes of HIF-1 such as vascular endothelial growth factor-A (VEGF-A), glucose transporter-1, and carbonic anhydrase IX (CAIX) [17–20].

HIF-1α is degraded rapidly under normoxic conditions, while under hypoxic conditions, HIF-1α is stabilized and can dimerize with HIF-1β to form the HIF-1 protein complex [21, 22]. Although HIF-1α is strongly regulated by the oxygen tension in tumors and HIF-1 has multiple target genes promoting radiation resistance and malignant progression [23], the potential of HIF-1α as a biomarker of hypoxia and hypoxia-induced tumor aggressiveness in cervical carcinoma is controversial [24–26]. High HIF-1α expression correlated significantly with low oxygen tension and high level of binding of the exogenous hypoxia marker pimonidazole in one study [25], whereas another study showed no correlation between HIF-1α expression and oxygen tension or hypoxic fraction [26]. Furthermore, a significant correlation between the outcome of radiation therapy and the expression of HIF-1α was reported in one investigation [24], whereas HIF-1α expression was found to have no significant prognostic impact in two other investigations [25, 26].

Among the target genes of HIF-1, CAIX seems to have the greatest potential as a biomarker of tumor hypoxia in cervical carcinoma [19, 27, 28]. CAIX appears to be strictly regulated by hypoxia via HIF-1α [27] and shows strong up-regulation in hypoxic regions of tumors of the cervix [19]. Moreover, high level of CAIX in cervical tumors has been shown to be associated with lymph node metastasis [29] and to predict for poor overall and metastasis-free survival rates after radiation therapy [19]. However, although significant correlations have been found between CAIX expression and tumor hypoxia as measured with polarographic electrodes [19], immunohistochemical comparisons of CAIX expression and pimonidazole binding have yielded conflicting results [30, 31]. Olive et al. [30] compared the CAIX and pimonidazole immunostaining in tumor sections from 18 cervical cancer patients and observed excellent colocalization and a strong correlation between the CAIX-positive and pimonidazole-positive tissue fractions, whereas in a similar study involving 42 patients, Airley et al. [31] did not find a significant correlation between CAIX expression and the level of pimonidazole binding.

The contradictory reports on the potential of HIF-1α and CAIX as endogenous markers of tumor hypoxia in cervical carcinoma may have multiple explanations, including different methods of measuring hypoxia, different methods of detecting HIF-1α and CAIX expression, and biological differences between the tumor cohorts subjected to investigation [28]. Mayer et al. [32] have discouraged the use of the expression ‘endogenous hypoxia markers’ and argue that the concept itself is unrealistic in a heterogeneous clinical setting, primarily because HIF-1α stabilization and the subsequent activation of HIF-1 target genes may be influenced by hypoxia-independent mechanisms.

Preclinical studies of human tumor xenografts have the advantage to clinical investigations that the experimental conditions can be controlled easily, and furthermore, that multiple copies of a patient’s tumor can be examined. The individual tumors of a xenograft line have the same genetic background, but may have significantly different physiological microenvironments due to the stochastic processes involved in tumor angiogenesis. The purpose of the present work was to challenge the view that HIF-1α and CAIX may be useful biomarkers of tumor hypoxia and hypoxia-induced tumor aggressiveness in cervical carcinoma by searching for possible associations between HIF-1α expression, CAIX expression, fraction of hypoxic tissue, and lymph node metastasis in tumors of two human cervical carcinoma xenograft lines. Pimonidazole was used as an exogenous hypoxia marker, and the CK-160 and TS-415 lines were selected for the study because previous investigations have revealed that individual tumors of these lines may differ substantially in hypoxic fraction and metastatic propensity [33–36].

## Methods

### Preclinical tumor models

CK-160 and TS-415 human cervical carcinoma xenografts growing in adult female BALB/c *nu*/*nu* mice were used as tumor models [33, 35]. Tumors were initiated from cell lines cultured in RPMI-1640 (25 mmol/l HEPES and l-glutamine) medium supplemented with 13 % bovine calf serum, 250 mg/l penicillin, and 50 mg/l streptomycin. Approximately 5.0 × 10^5^ cells in 10 μl of Hanks’ balanced salt solution were inoculated in the gastrocnemius muscle. Twenty CK-160 and 16 TS-415 tumors with volumes of 600–800 mm^3^ were included in the study. Animal care and experimental procedures were approved by our institution (Committee on Research Animal Care) and the Norwegian Animal Research Authority, and were performed in accordance with the Interdisciplinary Principles and Guidelines for the Use of Animals in Research, Marketing, and Education (New York Academy of Sciences, New York, NY, USA).

The CK-160 and TS-415 cell lines were established in our laboratory from pelvic lymph node metastases of patients admitted to the Norwegian Radium Hospital for the treatment of FIGO stage IIB squamous cell carcinoma of the uterine cervix [33]. The metastatic pelvic lymph nodes were surgically excised from the patients prior to radiation therapy. The use of metastatic lymph node tissue for establishing cervical carcinoma cell lines for experimental purposes was approved by our institution (Committee for Medical and Health Research Ethics). Written informed consent was obtained from the donors of metastatic lymph nodes.

### Tumor hypoxia and expression of HIF-1α and CAIX

Hypoxic tumor tissue, HIF-1α expression, and CAIX expression were detected by immunohistochemistry [37]. Pimonidazole [1-[(2-hydroxy-3-piperidinyl)-propyl]-2-nitroimidazole], injected as described previously, was used as an exogenous marker of tumor hypoxia [38]. An anti-pimonidazole rabbit polyclonal antibody (Professor Raleigh, University of North Carolina, Chapel Hill, NC, USA), an anti-HIF-1α rabbit polyclonal antibody (Abcam, Cambridge, UK), or an anti-CAIX rabbit polyclonal antibody (Abcam) was used as primary antibody. Diaminobenzidine was used as chromogen, and hematoxylin was used for counterstaining. Histological sections were prepared from three regions of each tumor. In each region, adjacent sections were stained with hematoxylin and eosin (HE) or immunostained for hypoxia, HIF-1α expression, or CAIX expression. Hypoxic fraction (HF_Pim_), HIF-1α positive fraction (PF_HIF-1α_), and CAIX positive fraction (PF_CAIX_), defined as the area fraction of the non-necrotic tissue staining positive for pimonidazole, HIF-1α, or CAIX, respectively, were assessed by image analysis [39].

### Metastasis

Spontaneous metastasis was studied as described in detail elsewhere [33, 36]. Briefly, after the primary tumor was resected for immunohistochemical analyses, the mice were examined for external lymph node metastases in the inguinal, axillary, interscapular, and submandibular regions and internal lymph node metastases in the abdomen and mediastinum. The presence of metastatic growth in enlarged lymph nodes was confirmed by histological examinations.

### Statistical analysis

The Pearson product moment correlation test was used to search for correlations between parameters. Statistical comparisons of data were carried out with the Student *t* test when the data complied with the conditions of normality and equal variance. Under other conditions, comparisons were carried out by nonparametric analysis using the Mann-Whitney rank-sum test. The Kolmogorov-Smirnov method was used to test for normality. Probability values of *P* < 0.05, determined from two-sided tests, were considered significant. The statistical analysis was performed by using the SigmaStat statistical software (SPSS Science, Chicago, IL, USA).

## Results

**Fig. 1:**
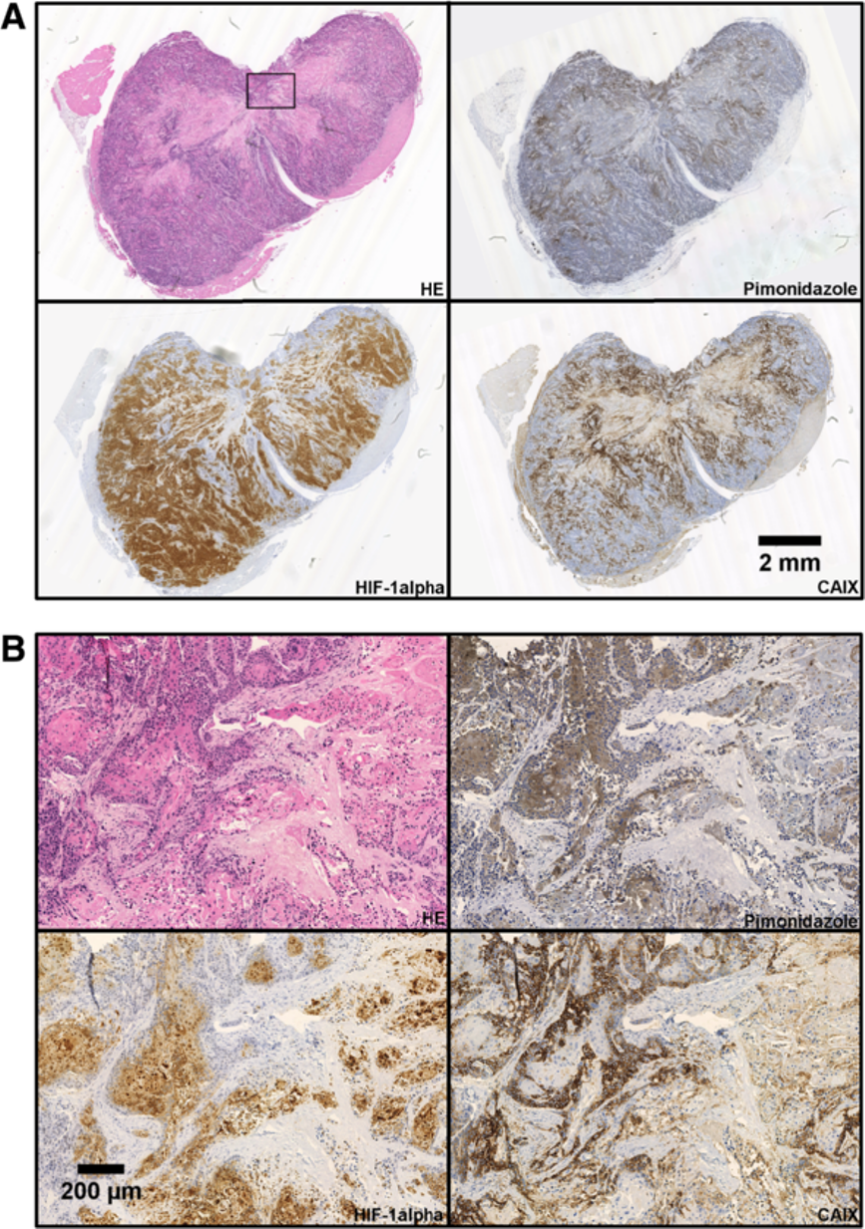
Histological appearance of CK-160 cervical carcinoma xenografts. Adjacent tumor sections stained with HE or immunostained for pimonidazole, HIF-1α, or CAIX shown at low (**a**) and high (**b**) magnification. The high magnification images refer to the squared box in (**a**)

**Fig. 2:**
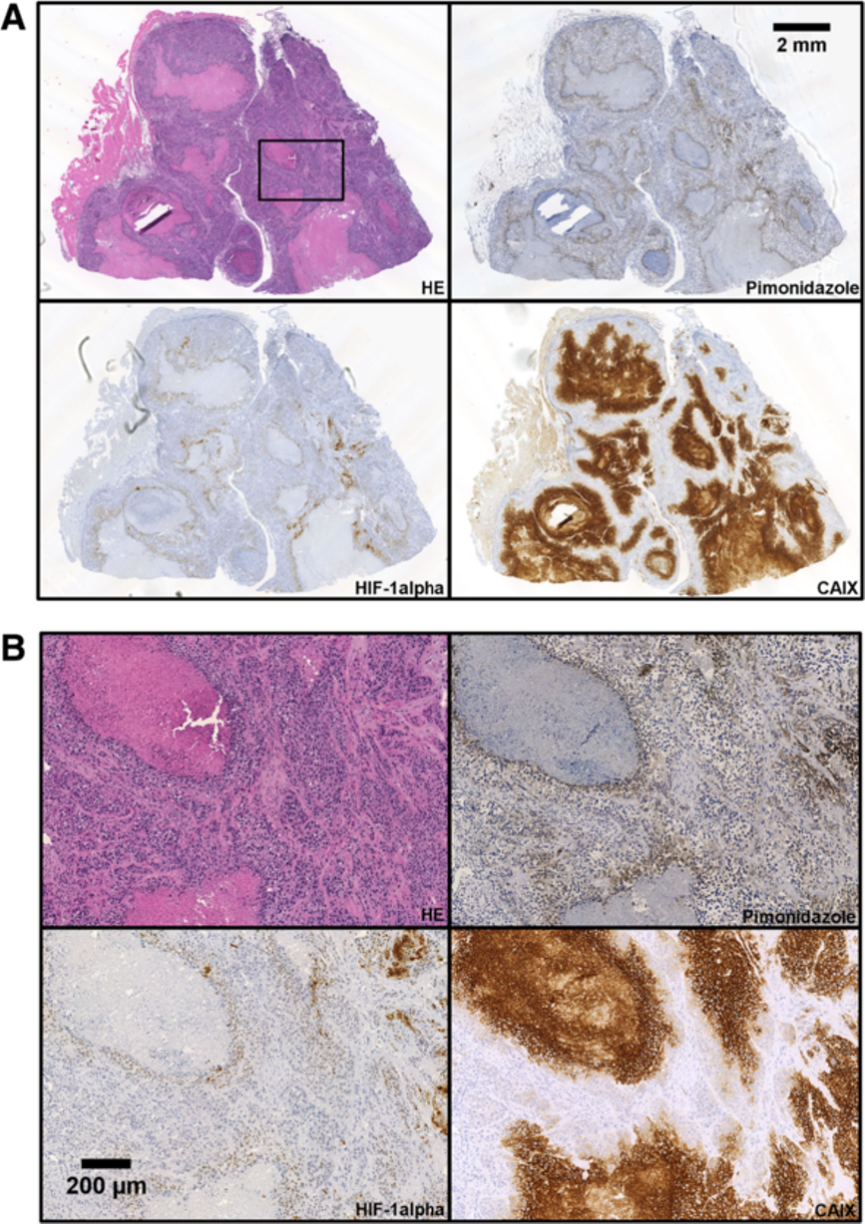
Histological appearance of TS-415 cervical carcinoma xenografts. Adjacent tumor sections stained with HE or immunostained for pimonidazole, HIF-1α, or CAIX shown at low (**a**) and high (**b**) magnification. The high magnification images refer to the squared box in (**a**)

Low and high magnification images of the histological appearance of a representative CK-160 tumor and a representative TS-415 tumor are presented in Figs. 1 and 2, respectively. The images refer to a HE section and adjacent sections stained for pimonidazole, HIF-1α, or CAIX. The tumors of both lines displayed considerable regions with necrotic tissue and nests of parenchymal cells interspersed with bands of connective tissue. Additionally, the CK-160 tumors produced significant amounts of keratin, frequently seen as secreted keratin pearls in central regions of tumor cell nests and in necrotic regions.

In CK-160 tumors, pimonidazole-positive cells and CAIX-positive cells were observed both adjacent to necroses and in some regions consisting predominantly of viable tissue, while strong staining for HIF-1α was seen throughout the tumor tissue (Fig. 1a). Examinations at high magnification revealed that pimonidazole and HIF-1α were localized primarily in the keratinizing centers of tumor cell nests, whereas CAIX showed an almost diametrical staining pattern with expression only in the periphery of these nests (Fig. 1b).

In TS-415 tumors, the pimonidazole staining pattern was consistent with staining of chronically hypoxic tissue, with bands of positive cells surrounding necrotic regions and a few small foci of positive cells in tissue regions without necroses (Fig. 2a). The expression of HIF-1α largely mirrored this pattern (Fig. 2a), but HIF-1α-positive cells did not extend as far from necrotic tissue as those positive for pimonidazole (Fig. 2b). The expression of CAIX was also associated with necrosis (Fig. 2a), but with positive cells extending far from necrotic regions (Fig. 2b).

**Fig. 3:**
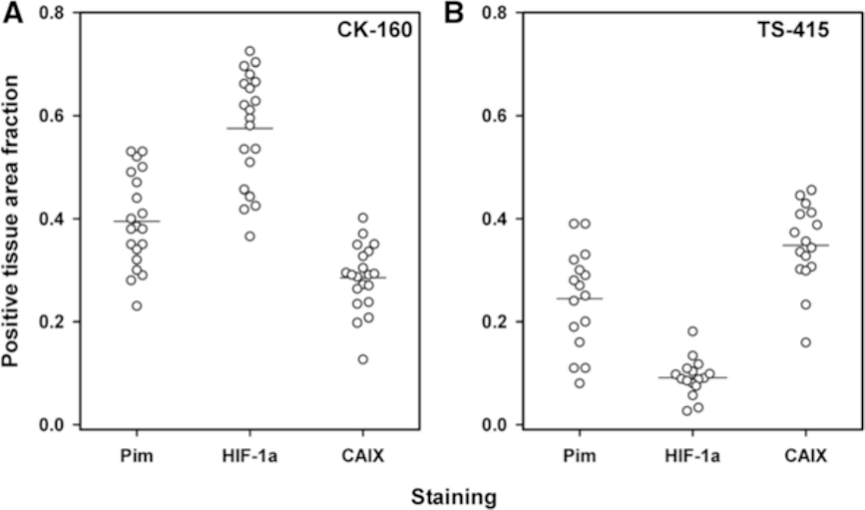
Pimonidazole and endogenous hypoxia markers. Tissue area fraction staining positive for pimonidazole, HIF-1α, or CAIX in CK-160 (**a**) and TS-415 (**b**) cervical carcinoma xenografts. Points, individual tumors; horizontal lines, mean values

**Fig. 4:**
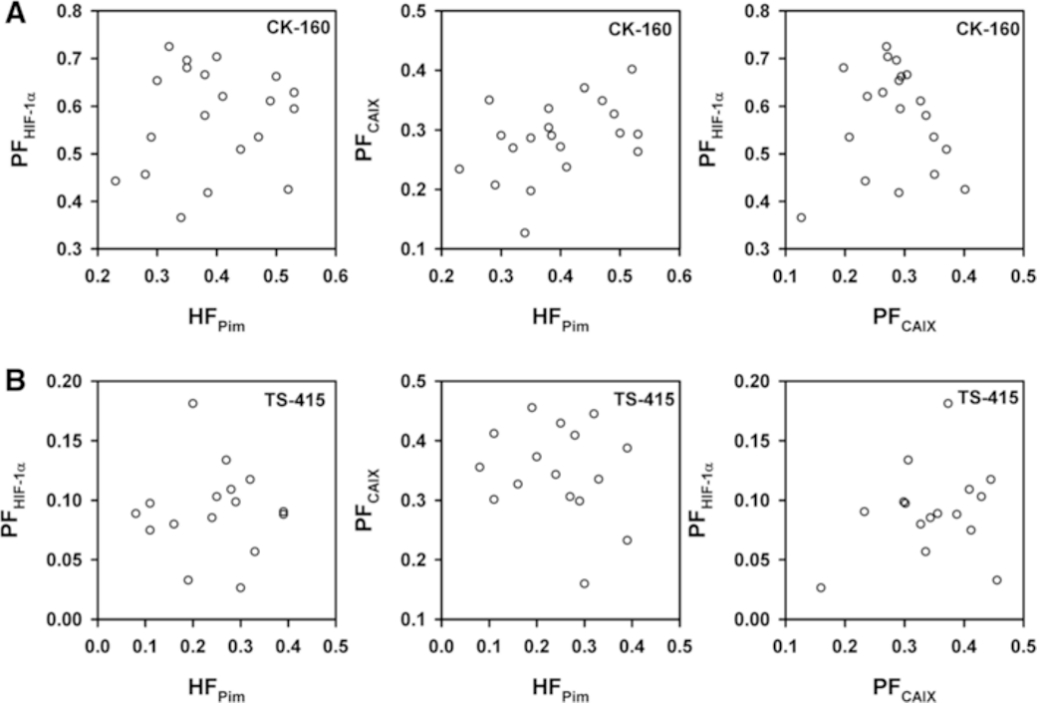
Endogenous hypoxia markers and tumor hypoxia. PF_HIF-1α_ *versus* HF_Pim_, PF_CAIX_ *versus* HF_Pim_, and PF_HIF-1α_ *versus* PF_CAIX_ for CK-160 (**a**) and TS-415 (**b**) cervical carcinoma xenografts. Points, individual tumors

Quantitative analyses showed that HF_Pim_, PF_HIF-1α_, and PF_CAIX_ differed substantially among individual CK-160 tumors (Fig. 3a) and individual TS-415 tumors (Fig. 3b). HF_Pim_ ranged from 0.23 to 0.53 (CK-160) and from 0.08 to 0.39 (TS-415), and was significantly higher in the CK-160 tumors than in the TS-415 tumors (*P* < 0.0001). PF_HIF-1α_ varied from 0.37 to 0.73 (CK-160) and from 0.03 to 0.18 (TS-415), and was also significantly higher in the CK-160 tumors than in the TS-415 tumors (*P* < 0.0001). In contrast, PF_CAIX_ differed from 0.13 to 0.40 (CK-160) and from 0.16 to 0.46 (TS-415), and was significantly lower in the CK-160 tumors than in the TS-415 tumors (*P* = 0.012). Interestingly, PF_HIF-1α_ was significantly higher than HF_Pim_ (*P* < 0.0001) and PF_CAIX_ was significantly lower than HF_Pim_ (*P* < 0.0001) in the CK-160 tumors, whereas in the TS-415 tumors, PF_HIF-1α_ was significantly lower than HF_Pim_ (*P* < 0.0001) and PF_CAIX_ was significantly higher than HF_Pim_ (*P* = 0.0022). Furthermore, there was no correlation between PF_HIF-1α_ and HF_Pim_, between PF_CAIX_ and HF_Pim_, or between PF_HIF-1α_ and PF_CAIX_ in any of the lines (*P* > 0.05, Fig. 4).

**Fig. 5:**
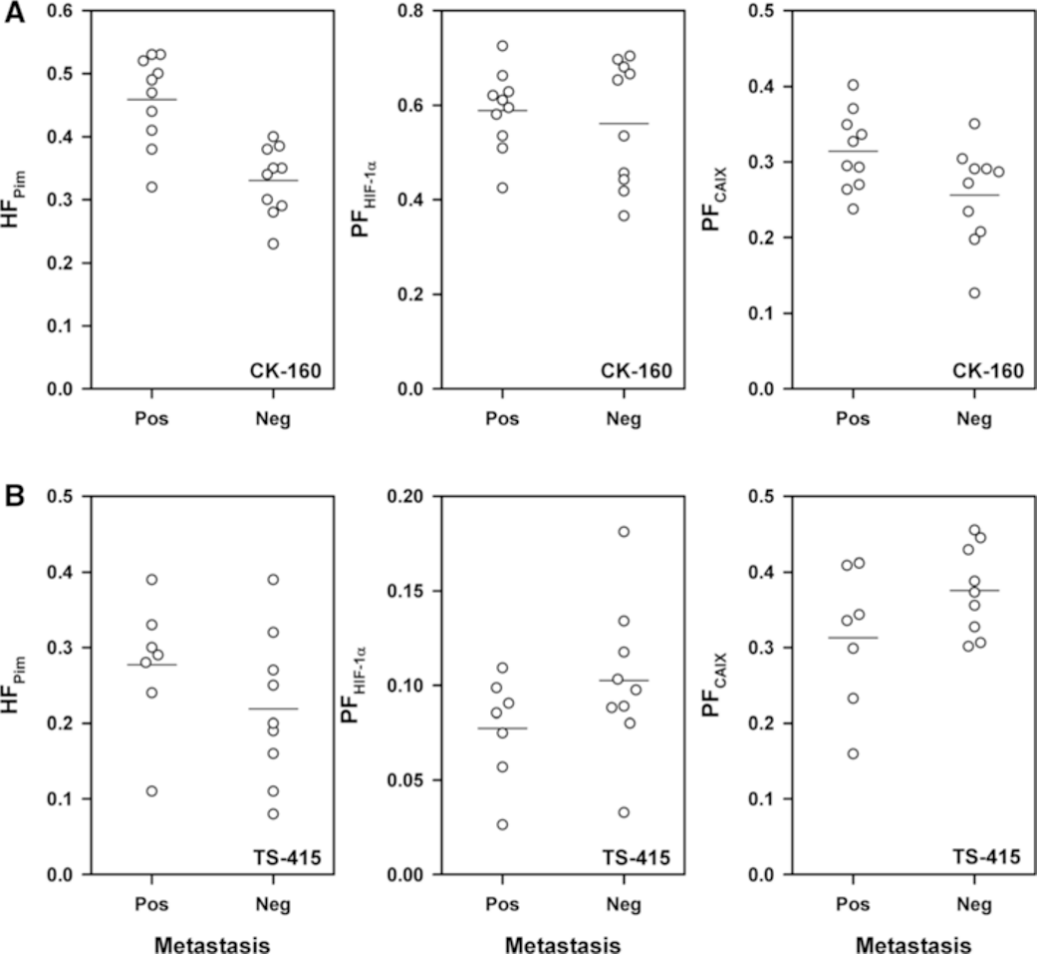
Endogenous hypoxia markers and tumor metastasis. HF_Pim_, PF_HIF-1α_, or PF_CAIX_ in metastatic and nonmetastatic CK-160 (**a**) and TS-415 (**b**) cervical carcinoma xenografts. Points, individual tumors; horizontal lines, mean values

Lymph node metastases were detected in 10 of the 20 mice bearing CK-160 tumors and in 7 of the 16 mice bearing TS-415 tumors. In the CK-160 line, the primary tumors of the metastasis-positive mice showed significantly higher HF_Pim_ (*P* = 0.0002) and significantly higher PF_CAIX_ (*P* = 0.039) than those of the metastasis-negative mice, but did not differ from the primary tumors of the metastasis-negative mice in PF_HIF-1α_ (*P* > 0.05, Fig. 5a). The metastatic and nonmetastatic TS-415 tumors were not significantly different in any of these three parameters (*P* > 0.05, Fig. 5b). Moreover, none of the parameters were found to differ between the metastatic and nonmetastatic tumors when the data from the two tumor lines were analyzed together (*P* > 0.05 for HF_Pim_, PF_HIF-1α_, and PF_CAIX_).

## Discussion

Tumor hypoxia represents a significant obstacle in the management of locally advanced carcinoma of the uterine cervix, by increasing malignancy and inducing treatment resistance [5–7, 12–15]. A major player in the hypoxia response is HIF-1α, with its downstream target genes involved in cell survival, metabolism, angiogenesis, and metastasis [17–23]. Based on a number of clinical studies, several investigators have promoted the view that the expression of HIF-1α and its target genes may be useful biomarkers of tumor hypoxia, aggressiveness, and radiation resistance in cervical carcinoma [16, 18, 19, 24, 25, 29, 30]. In the present preclinical study, we challenged this view by searching for correlations between tumor hypoxia and the expression of HIF-1α and CAIX in CK-160 and TS-415 cervical carcinoma xenografts.

A significant advantage of preclinical investigations over clinical investigations is that the measurement conditions can be kept unchanged during a study. A major disadvantage of preclinical studies is that experimental tumors may not necessarily be valid models of human cancer. The CK-160 and TS-415 tumors used in this study have some limitations as models of primary tumors of the uterine cervix. First, the tumors were transplanted ectopically to the gastrocnemius muscle, and ectopic tumors may be inferior to orthotopic tumors as models of cancer in humans. Second, the CK-160 and TS-415 cell lines were established from lymph node metastases, and the biological properties of lymph node metastases may not be equal to those of primary tumors. It should also be noticed that only the parenchyma of xenografted tumors is of human origin whereas the stroma is recruited from the host and thus is of murine origin. Furthermore, immune-deficient animals like BALB/c *nu*/*nu* mice do not show a normal inflammatory response, and the stromal conditions in tumors in such hosts may not necessarily be representative for human tumors.

On the other hand, previous investigations have revealed that CK-160 and TS-415 tumors retain several biological properties of the donor patients’ primary tumors when transplanted intramuscularly in BALB/c *nu*/*nu* mice, including response to radiation therapy, histological appearance, and metastatic pattern and propensity [33, 34]. These observations suggest that CK-160 and TS-415 xenografts should be useful models for investigating the potential of HIF-1α and CAIX as biomarkers of hypoxia and metastasis in tumors of the cervix.

Cervical carcinomas generally show significant intratumor heterogeneity in oxygenation [6, 7, 13, 14], and oncogenic mutations induced by fluctuating hypoxia or other factors may lead to the development of multiple cell subpopulations [16, 26]. Intratumor heterogeneity is a problem in all biopsy-based methods being used for characterizing malignant tumors and identifying hypoxia biomarkers. Distinctly different cell subpopulations were probably not a problem in this study since the CK-160 and TS-415 tumors were initiated from established cell lines showing no clonal heterogeneity. However, both lines showed significant intratumor heterogeneity in pimonidazole, HIF-1α, and CAIX staining. To reduce the possibility of erroneous conclusions caused by this heterogeneity, we subjected three different regions of each tumor to immunostaining and histological examinations.

The degrees of colocalization between pimonidazole-positive tissue and HIF-1α- or CAIX-positive tissue were poor in tumors of both lines. In the CK-160 tumors, PF_HIF-1α_ was higher and PF_CAIX_ was lower than HF_Pim_, whereas PF_HIF-1α_ was lower and PF_CAIX_ was higher than HF_Pim_ in the TS-415 tumors. Furthermore, HF_Pim_ did not correlate with PF_HIF-1α_ or PF_CAIX_ in any of the tumor lines. Because HIF-1α and CAIX are poor biomarkers of hypoxia in xenografted tumors having the same genetic background but differing in the extent of hypoxia, it is unlikely that HIF-1α and CAIX can be useful biomarkers of hypoxia in the clinical setting. Our study thus provides strong support to the view of Mayer et al. [32] that HIF-1α and its target genes should not be referred to as endogenous hypoxia markers.

Lack of correlation between the extent of pimonidazole staining and the extent of HIF-1α or CAIX staining in cervical carcinoma may be a consequence of both tumor-specific conditions and tumor-independent mechanisms. The tumor-independent mechanisms include differences in the duration and level of hypoxia required for formation of pimonidazole adducts and expression of HIF-1α and CAIX proteins as well as differences in the half-lives of the adducts and the proteins [40, 41]. Moreover, the extent of HIF-1α and CAIX staining can be influenced by the proliferation status of the hypoxic tumor cells [40] and their metabolic and physiological microenvironments, including the level of reactive oxygen and nitrogen species, lowered extracellular pH and glucose and bicarbonate deprivation, and the concentrations of some growth factors and cytokines [28, 32, 41, 42]. The fact that significant correlations between HF_Pim_ and PF_HIF-1α_ or PF_CAIX_ were not found in any of the two tumor lines included in the present study underscores that the influence of tumor-independent conditions is substantial.

The tumor-specific conditions include genetic changes leading to stabilization of HIF-1α under normoxic conditions, alterations of the HIF-1α degradation pathway, and activation of HIF-1 target genes by hypoxia-independent mechanisms [28, 32]. Several relevant genetic changes have been identified, including loss of function of tumor suppressor genes, for example the von Hippel-Lindau gene, and gain of function of oncogenes leading to PI3K/AKT/mTOR activity [43]. The importance of tumor-specific mechanisms is underscored by the observation that the ratios of PF_HIF-1α_ and PF_CAIX_ to HF_Pim_ were fundamentally different in CK-160 and TS-415 tumors.

The differences in the ratios of PF_HIF-1α_ and PF_CAIX_ to HF_Pim_ between the CK-160 and TS-415 lines reflected that the tumors of these lines showed fundamentally different staining patterns. The TS-415 tumors showed a perinecrotic staining pattern consistent with the classical view that HIF-1α is stabilized in hypoxic tissue and induces CAIX expression. The CK-160 tumors, in addition to showing a perinecrotic staining pattern, also showed staining in tissue regions far from necroses. Typically, the keratinizing centers of the cellular nests in CK-160 tumors showed significant staining for pimonidazole and HIF-1α, whereas a diametrical staining pattern was seen for CAIX with positive staining primarily in the peripheral layers of the nests. This staining pattern resembles the hypoxia staining pattern observed in moderately differentiated squamous cell carcinomas of the uterine cervix in humans [44–47]. In these clinical studies, positive staining for pimonidazole and the differentiation marker involucrin was seen primarily in the central regions of tissue nests [44, 45], whereas positive staining for the hypoxia-inducible proteins metallothionein (MT) and VEGF-A was detected primarily in the peripheral regions of the nests [46, 47]. These observations led to the suggestion that the differentiation of hypoxic cells in cervical carcinomas exerts collateral control on gene expression whereby the expression of hypoxia-inducible proteins like MT and VEGF-A is suppressed [45, 46]. In accordance with this suggestion, the localization of CAIX in CK-160 tumors indicates that the transcriptional status of CAIX is also altered by differentiation.

Some clinical investigations have revealed that high expression of HIF-1α and/or CAIX may be associated with high incidence of lymph node metastases and poor metastasis-free survival rates in cervical cancer patients treated with radiation therapy [17, 19, 24, 29]. These observations have led to the hypothesis that HIF-1α and CAIX may be useful biomarkers of the outcome of locally advanced cervical carcinoma even though the expression of these proteins may not be strongly related to the extent of tumor hypoxia. The present study does not support this view. First, previous investigations have revealed that lymph node metastasis is associated with extensive hypoxia in CK-160 xenografts [33, 35], and this was confirmed in the study reported here. PF_HIF-1α_ was not associated with lymph node metastasis in CK-160 tumors, whereas PF_CAIX_ was slightly higher in the metastatic than in the nonmetastatic tumors, but did not discriminate as well as HF_Pim_. Second, there was no association between HF_Pim_ and lymph node metastasis in TS-415 tumors, in accordance with previous investigations [33, 36]. The metastatic and nonmetastatic TS-415 tumors did not differ significantly in either PF_HIF-1α_ or PF_CAIX_, but both PF_HIF-1α_ and PF_CAIX_ tended to be higher in the metastasis-negative than in the metastasis-positive tumors, in contrast to the hypothesis described above. Furthermore, neither PF_HIF-1α_ nor PF_CAIX_ was found to be associated with lymph node metastasis when the data from the CK-160 and TS-415 tumors were pooled. Taken together, these observations suggest that high expression of HIF-1α or CAIX cannot be clinically useful biomarkers of poor outcome in squamous cell carcinoma of the uterine cervix.

## Conclusions

The staining patterns of pimonidazole, HIF-1α, and CAIX in CK-160 tumors differ from the corresponding staining patterns in TS-415 tumors, and there is no correlation between HF_Pim_ and PF_HIF-1α_ or PF_CAIX_ in any of these human cervical carcinoma xenograft lines. Moreover, lymph node metastasis is not a result of high expression of HIF-1α or CAIX in CK-160 and TS-415 tumors. The individual tumors of a xenograft line have identical genetic background in contrast to individual tumors in cervical cancer patients, and consequently, the observations reported here show that it is highly unlikely that the expression of HIF-1α or CAIX can be useful biomarkers of tumor hypoxia and/or outcome of chemoradiotherapy in locally advanced cervical carcinoma.

## Abbreviations

CAIX: Carbonic anhydrase IX
HE: Hematoxylin and eosin
HIF-1α: Hypoxia inducible factor-1α, HF_Pim_, Hypoxic fraction, i.e., fraction of non-necrotic tissue staining positive for pimonidazole
MT: Metallothionein
PF_CAIX_: Positive fraction for CAIX, i.e., fraction of non-necrotic tissue staining positive for CAIX
PF_HIF-1α_: Positive fraction for HIF-1α, i.e., fraction of non-necrotic tissue staining positive for HIF-1α
VEGF-A: Vascular endothelial growth factor-A
